# Recurrent pulmonary embolism and azygos vein thrombosis during varenicline therapy for smoking cessation

**DOI:** 10.1093/omcr/omaf164

**Published:** 2025-09-28

**Authors:** Stanley Kim, BreeAnna Carlson, Kevin Chen, Frank Sabatelli

**Affiliations:** Division of Hematology and Oncology, Kern Medical Center, 1700 Mount Vernon Avenue, Bakersfield, CA 93306, Unites States; Loyola University Medical Center, Loyola University Chicago Stritch School of Medicine, 2160 S. First Avenue, Maywood, IL 60153, Unites States; Medical student, Western University of Health Science-College of Osteopathic Medicine of the Pacific, 200 Mullins Drive, Lebanon, OR 97355-3983, United States; Medical student, Western University of Health Science-College of Osteopathic Medicine of the Pacific, 309 E. Second Street, Pomona, CA 91766-1854, United States; Department of Radiology, Kern Medical Center, 1700 Mount Vernon Avenue, Bakersfield, CA 93306, United States

**Keywords:** pulmonary embolism, varenicline, venous thromboembolism, azygos vein thrombosis, smoking cessation

## Abstract

Venous thromboembolism (VTE) associated with varenicline is extremely rare. We present the case of a young woman who experienced two distinct episodes of pulmonary embolism (PE) while taking varenicline. The patient developed her first episode of PE two months after varenicline use. Four years later, she experienced a second episode of PE and azygos vein thrombosis three weeks after restarting varenicline. Both episodes occurred in the absence of any identifiable provoking factors. Her family history of VTE was negative, and an evaluation of hypercoagulability revealed no abnormalities. We report the first documented case of recurrent PE and the first case of azygos vein thrombosis associated with varenicline use. This case may suggest a potential association between varenicline use and VTE. Clinicians should assess the VTE risk and closely monitor patients taking varenicline.

## Introduction

Varenicline is one of the most effective medications used for smoking cessation [1]. As a partial agonist of α4β2 nicotinic acetylcholine receptors (nAChRs), varenicline partially stimulates these receptors in the basal mesolimbic dopamine system, mimicking the effects of nicotine but at a lower intensity (50% of the maximal effect of nicotine). This action reduced cravings and withdrawal symptoms [2].

There have been concerns regarding the potential association of varenicline with an increased risk of cardiovascular events, such as angina or myocardial infarction. A randomized study showed a statistically insignificant, small increase in cardiovascular events in patients with pre-existing cardiovascular conditions [2]. However, another randomized study found that varenicline did not increase cardiovascular events in the general population [3].

No clinical studies have demonstrated an association between varenicline use and venous thromboembolism (VTE). The available literature, including the adverse reaction reports of the U.S. Food and Drug Administration (FDA), does not list VTE as an adverse effect of varenicline [4]. Only one reported case has suggested a probable association between varenicline use and pulmonary embolism (PE) [5]. Here, we present the first documented case of recurrent PE and the first case of azygos vein thrombosis associated with varenicline use.

## Case report

### The first episode of PE in august 2020

**Figure 1:**
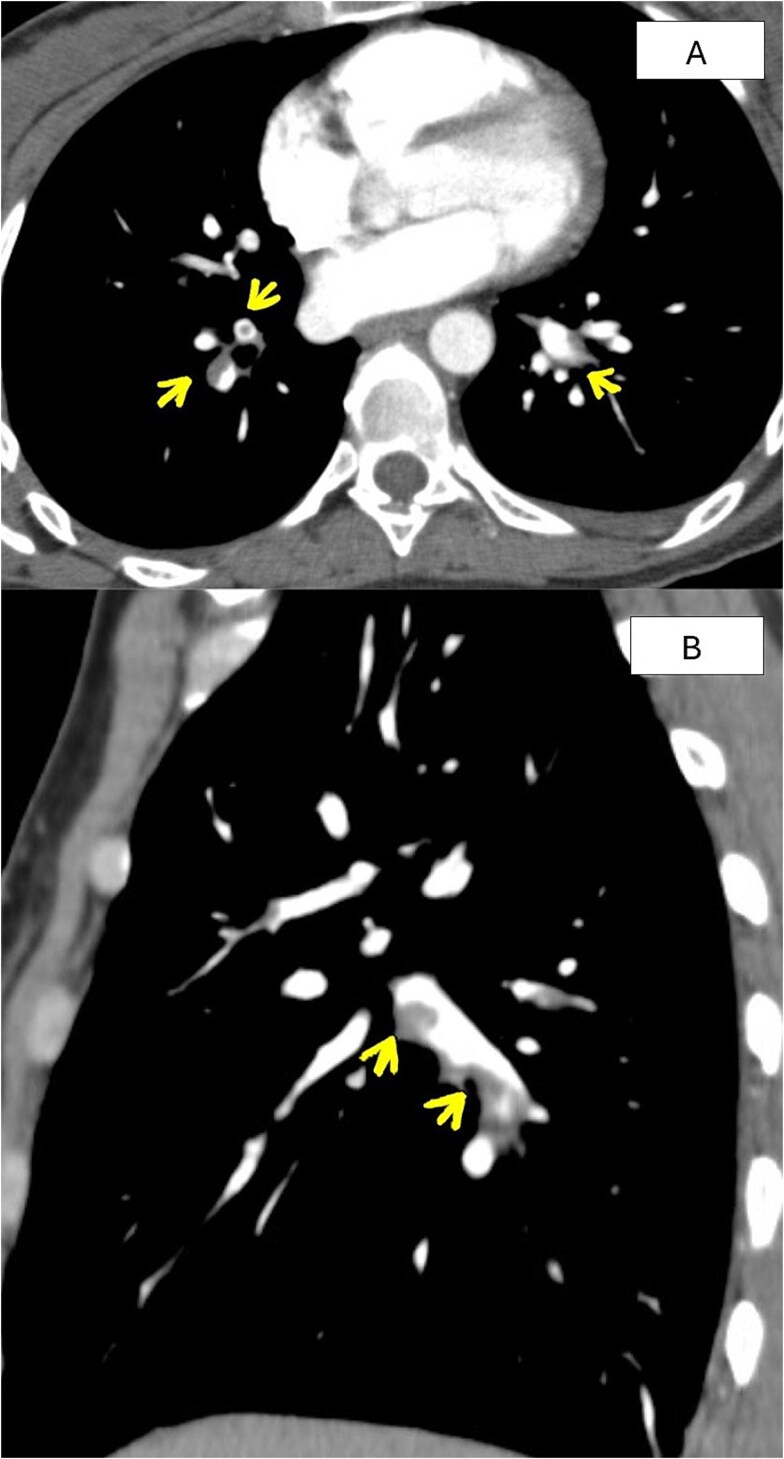
(A) CT angiogram (CTA) chest (august 2020) showing multiple partial thrombi in the right subsegmental left segmental branches (arrowheads). (B) Sagittal 2-dimensional reconstruction CTA chest (august 2020) reveals mural partial thrombosis. Filling defects in the segmental and subsegmental right pulmonary artery branches (arrowheads).

A 36-year-old woman with a history of tobacco use disorder presented to the emergency department (ED) with acute left-sided chest pain and shortness of breath. She had a 10-pack-year smoking history and was still smoking 10 cigarettes per day. Otherwise, she was healthy, with no history of obesity, hypertension, diabetes mellitus, coronary artery disease, or stroke. She had not experienced any recent illnesses, injuries, surgeries, or COVID-19 infection. She was not taking hormonal medications, including oral contraceptives or hormonal intrauterine devices. Her only reported medication was varenicline, which she had started two months earlier for smoking cessation. She had no family history of clotting disorders or premature cardiovascular diseases. On physical examination, her blood pressure was 96/68 mmhg, respiratory rate 20 breaths/min, heart rate 96 beats/min, and oxygen saturation was 96% on room air. Her breath sounds were clear, and the left-sided chest was non-tender. Laboratory results showed an elevated D-dimer level of 434 ng/ml (normal 0-230 ng/ml), while all other laboratory findings were unremarkable. Computed tomography angiography (CTA) of the chest revealed filling defects in the pulmonary arterial branches bilaterally (Fig. 1A and B). Vascular duplex ultrasound (US) of both lower extremities was negative for deep vein thrombosis (DVT). She was initially treated with subcutaneous enoxaparin injections and later transitioned to apixaban which was continued for six months. After the discontinuation of the apixaban, outpatient hypercoagulability testing returned normal results (Table 1). A follow- up CTA of the chest revealed resolution of the PE.

**Table 1:** Hypercogulobility tests and results.

Name of test	Result	Normal value
Factor V Leiden	Negative	Negative
Protein C activity	177%	65-135%
Protein S activity	77%	60-150%
Antithrombin -III activity	140%	80-120%
Prothrombin Factor-II mutation	Negative	Negative
Anti-cardiolipin IgG antibody	<2.0 GPL u/ml	<15 GPL u/ml
Anti-cardiolipin IgM antibody	<2.0 MPL u/ml	<12.5 MPL u/ml
β-2 glycoprotein 1 IgG antibody	<2.0 SGU u/ml	<20 SGU u/ml
β-2glycoprotein 1 IgM antibody	<2.0 SMU u/ml	<20 SMU u/ml
Homocysteine	6.6 mcmol/l	<15 mcmol/l
Prothrombin time (PT)	11.6 s	11-13.5 s
Partial thromboplastin time (PTT)	26.5 s	25-35 s

### The second episode of PE 4 years later in august 2024

**Figure 2:**
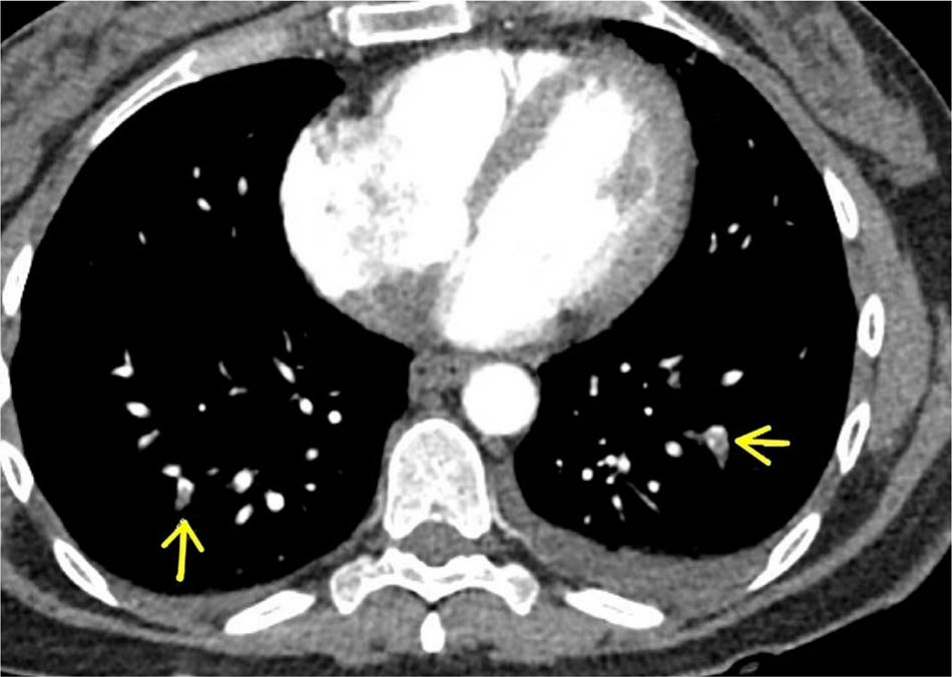
CTA chest (august 2024) showing bilateral subsegmental pulmonary artery embolism (arrows).

**Figure 3:**
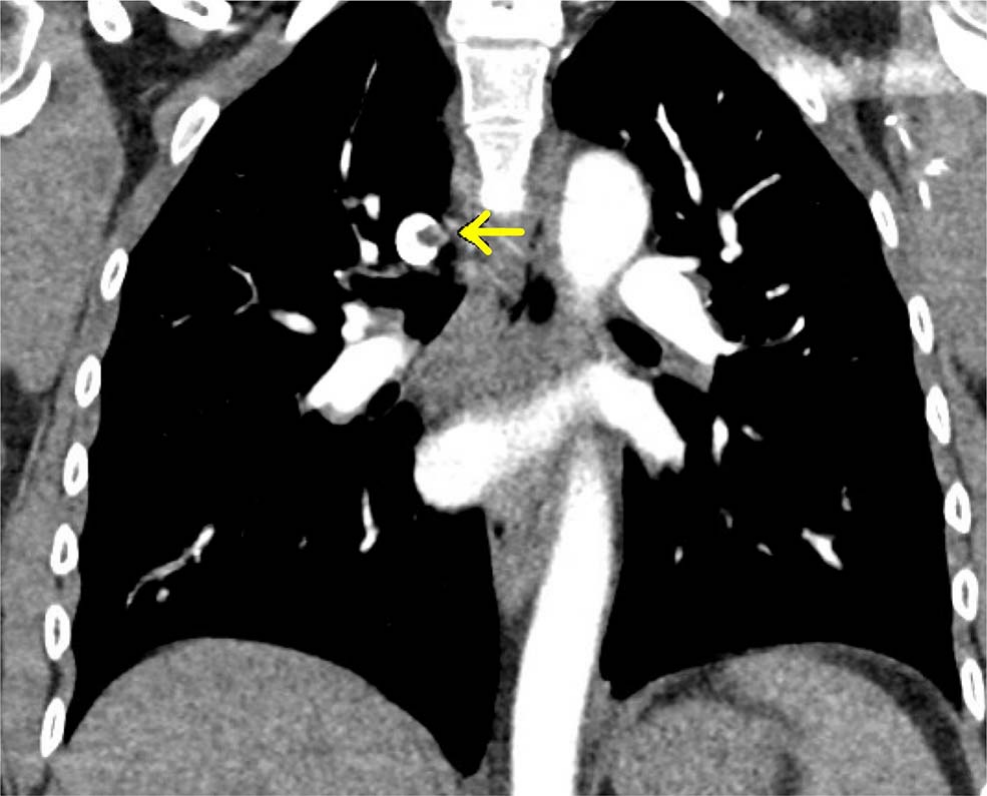
CTA chest (august 2024) demonstrating azygos vein medial wall thrombosis.

The same female, now 41-year-old, presented to the ED with left-sided chest pain that had been worsening over the past four days. Three weeks earlier, she had visited her primary care physician for smoking cessation assistance and was prescribed the varenicline which she had been taking for under three weeks before the ED visit. The patient was not certain about the specific brand of varenicline she took. Laboratory tests, electrocardiography, troponin level, and chest radiography findings were unremarkable. A CTA revealed filling defects in the subsegmental arteries bilaterally (Fig. 2). In addition, a large filling defect was found in the azygos vein, which was consistent with azygos vein thrombosis (Fig. 3). No DVT was observed in either leg on US. The patient was successfully treated using apixaban. Again, no identifiable provoking factors were identified.

## Discussion

There is no clear evidence that directly links varenicline to an increased risk of VTE. Neither the FDA nor the European Medicines Agency (EMA) has listed VTE as a significant adverse effect of varenicline [4, 6]. Only one prior case of PE, possibly induced by varenicline, has been reported [5]. Our patient was a young, active woman with no personal or family history of VTE, and negative hypercoagulability screening tests (Table 1). No factors provoking VTE were identified. The only known risk factor for VTE in our patient was cigarette smoking. While smoking is associated with an increased risk of both arterial and venous thromboembolisms [7], a prospective study found that the risk of VTE increases primarily in heavy smokers (>20 pack-year history) and in the presence of smoking-related disease or other predisposing factors [8]. Our patient was not a heavy smoker (10 pack-year history) and had neither smoking-attributable disease nor any other recognized risk factors. Therefore, it is unlikely that PEs were solely related to smoking. This case strongly suggests a causal relationship between varenicline use and PE.

Varenicline’s primary pharmacological action is as a partial agonist of α4β2 nAChRs. It also acts as a partial agonist of α7 nAChRs, which are highly expressed in the brain and play a role in cognition, memory, and reward pathways. A significant reduction in α7 nAChRs in the brain, particularly in the hippocampus, has been reported in patients with Alzheimer’s disease and schizophrenia [9]. However, platelets also express α7 nAChRs, and stimulation of this receptor activates platelets and induces their aggregation [10]. Therefore, varenicline may potentiate thromboembolism development in smokers through its effect on platelet aggregation. If this hypothesis is confirmed, platelet function inhibitors, such as aspirin, may help mitigate the cardiovascular and thromboembolic risks associated with varenicline. A randomized study is required to validate this hypothesis. Nevertheless, the benefits of smoking cessation outweigh the small potential risk of thromboembolism associated with varenicline. Clinicians should carefully assess the VTE risk and monitor patients taking this medication.

## Conclusion

In this report, we present the first documented case of recurrent PE and azygos vein thrombosis occurring during varenicline therapy. This case highlights the importance of considering varenicline a potential contributor to venous thromboembolic events. Further research is warranted to investigate the mechanism, prevalence, and preventive measures of thromboembolism associated with varenicline.

## Consent

The patient consented for this case report including the images.

## Guarantor

There is no guarantor involved in this case report.
